# Efficacy of Compression Stockings in Prophylaxis of Lower Limb Lymphedema in Women Undergoing Treatment for Gynecological Malignancies: A Prospective Randomized Study

**DOI:** 10.3390/cancers17152530

**Published:** 2025-07-31

**Authors:** Joanna Kurpiewska-Pieniążek, Katarzyna Ochałek, Tomasz Grądalski, Andrzej Szuba

**Affiliations:** 1 Faculty of Health Sciences, Andrzej Frycz Modrzewski Krakow University, 30-705 Kraków, Poland; 2 St. Lazarus Hospice, 31-831 Kraków, Poland; k.ochalek@wp.pl (K.O.); tomgr@mp.pl (T.G.); 3 Department of Clinical Rehabilitation, University of Physical Culture, 31-571 Kraków, Poland; 4 Chair of Palliative Care, Medical Faculty, Andrzej Frycz Modrzewski Krakow University, 30-705 Kraków, Poland; 5 Department of Angiology and Internal Medicine, Wroclaw Medical University, 50-367 Wrocław, Poland; andrzej.szuba@umw.edu.pl

**Keywords:** lower limb lymphedema, gynecological cancer, compression therapy, prevention, quality of life

## Abstract

Background: Lower limb lymphedema (LLL) is a frequent complication after gynecological cancer treatment, with a significant impact on quality of life. Despite the common use of compression therapy in managing established lymphedema, its role in prevention remains insufficiently explored. Methods: In this prospective randomized study, 64 women treated for gynecological malignancies were assigned to either a compression group (CG) using medium-pressure stockings (23–32 mmHg) or a no-compression group (NCG). All participants received standard education and physical activity guidance. Limb volume, symptom burden, and quality of life were assessed over 12 months. Results: The incidence of LLL was significantly lower in the CG (3.4%) compared to the NCG (38%, *p* = 0.003). Compression use resulted in significant reductions in limb volume and symptom severity, as well as improved physical functioning. Compliance with compression therapy was high, and patients reported good comfort and usability. Conclusions: Medium-pressure compression stockings combined with education and physical activity are effective and well-tolerated in preventing LLL following gynecological cancer treatment.

## Simple Summary

Lower limb lymphedema (LLL) is a common yet underdiagnosed complication after gynecological cancer treatment that can significantly impair quality of life. This study evaluated the effectiveness of medium-pressure compression stockings, combined with education and physical activity, in preventing LLL in women after cancer surgery. The results showed a significant reduction in lymphedema incidence and symptom severity in the compression group compared to the controls. These findings support the use of compression therapy as a safe and effective preventive strategy.

## 1. Introduction

Lower limb lymphedema (LLL) is one of the most disabling and debilitating complications following inguinofemoral lymphadenectomy and pelvic radiotherapy [1]. The reported prevalence of LLL after treatment ranges from 5% to 58% [2]. Although the majority of cases occur within the first year post-treatment, studies have demonstrated that the risk of developing lymphedema persists long-term [3]. Notably, approximately 30% of LLL cases develop ≥10 years after treatment, with the highest incidence observed following vulvar cancer therapy [4].

Gynecological cancer-related lymphedema remains significantly under-recognized and under-treated, partly due to limited awareness among healthcare professionals and patients regarding early diagnosis and management of LLL, and partly due to the lack of standardized evaluation and diagnostic protocols [5]. Limb swelling leads to pain, heaviness, erythema, and a sensation of tightness. It is estimated that 26–67% of patients with gynecological cancer experience chronic fatigue or tiredness [6]. Fatigue is frequently reported among patients with lower limb lymphedema and interferes with physical functioning and daily activities such as housework, standing, and walking. Women affected by lymphedema are at increased risk of experiencing psychological symptoms including anxiety, depression, sleep disturbances, and fear of cancer recurrence [7], as well as recurrent infections such as dermatolymphadenitis.

Few studies have investigated physiotherapeutic interventions—such as manual lymphatic drainage, exercise programs combined with compression, or health education—for reducing the incidence of lymphedema [8,9,10]. Only a limited number of studies have evaluated the effects of low-pressure compression therapy (class I, 18–21 mmHg) in patients at risk of gynecological cancer-related lymphedema following treatment for malignant genital tumors. These studies suggest that the prophylactic use of compression stockings is both feasible and beneficial in this patient population [11,12]. However, data on the effectiveness of medium-pressure compression devices (23–32 mmHg) in reducing lymphedema incidence remain controversial. Observations from patients treated with inguinal lymphadenectomy for various malignancies have questioned the effectiveness of compression therapy in preventing LLL [13].

The optimal compression pressure for the prevention of lower limb lymphedema (LLL)—one that remains effective while maintaining long-term patient comfort and quality of life—remains a matter of ongoing debate. In particular, there is concern that prolonged use of compression therapy may negatively affect the quality of life of women who have successfully undergone cancer treatment. Therefore, further large-scale studies are warranted to clarify the role of compression therapy in reducing the incidence of LLL.

The aim of this study was to evaluate the effectiveness of medium-pressure compression stockings (23–32 mmHg) in reducing the incidence of lower limb lymphedema and associated symptoms, as well as to assess the comfort and tolerability of compression use over a 12-month follow-up period in women undergoing treatment for gynecological malignancies.

## 2. Materials and Methods

### 2.1. Participants

Between 2017 and 2020, a total of 620 patients were evaluated for surgical treatment of gynecological malignancies. Of these, 120 women met the eligibility criteria: age ≥ 18 years; diagnosis of cervical, uterine, endometrial, ovarian, or vulvar cancer with planned lymphadenectomy (routinely performed in accordance with tumor type and stage); or a BMI > 25.0 kg/m^2^ in cases of endometrial or ovarian cancer regardless of whether pelvic lymphadenectomy was planned.

Exclusion criteria included a history of limb edema, Eastern Cooperative Oncology Group (ECOG) performance status > 2, congestive heart failure, renal failure, hypothyroidism, leg ulceration, severe lower limb ischemia (Fontaine class ≥ 2), metastatic disease or recurrence, severe depression, and a history of other malignancies treated within five years prior to study enrollment.

Seventy-five patients who consented to participate were preoperatively randomized into two groups using a simple randomization method (random number generator): the compression group (CG), which received compression stockings, and the no-compression group (NCG), which received no prophylactic compression.

Eleven patients were excluded postoperatively for various reasons, resulting in a final sample of 64 women (n = 32 in CG; n = 32 in NCG).

The main reasons for withdrawal from the study included long travel distances, refusal to use compression stockings for 12 months, postoperative complications, and death.

**Figure 1:**
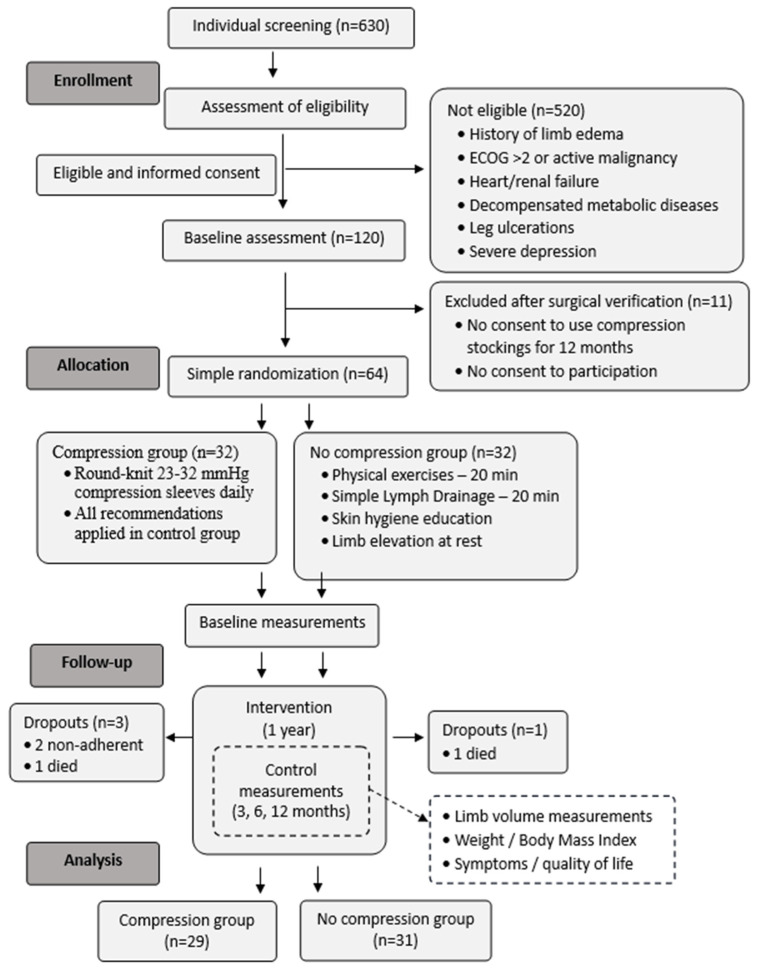
Flow chart regarding recruitment of study participants.

After the 12-month intervention period, data from 60 women were analyzed (29 in CG; 31 in NCG) (Figure 1).

The two groups were comparable with respect to baseline characteristics, including body mass index (BMI), tumor localization, type of surgery, adjuvant therapy, and initial limb volume measurements (Table 1). Data are presented as n for categorical variables and as median (IQR) for continuous variables.

### 2.2. Intervention

Women in the CG were provided with individually fitted, round-knit thigh-high compression stockings exerting a pressure of 23–32 mmHg (class II, according to the RAL-GZG standard classification) worn on both lower limbs. The recommended daily wear time was approximately nine hours. Compression garments were selected based on circumference measurements taken in the morning and were all manufactured by the same company (Medi GmbH & Co. KG, Bayreuth, Germany). Compression shorts were not used in this study.

In both groups (CG and NCG), participants were encouraged to engage in regular physical activity, specifically walking for 20 min per day. In the CG, physical activity was performed while wearing the compression stockings.

All participants received comprehensive education regarding lymphedema prevention, including skin care, self-administered manual lymphatic drainage (simple lymphatic drainage, SLD), and general lifestyle recommendations. Proper skin hygiene included daily washing with hypoallergenic soap, careful drying—especially between the toes—and routine moisturizing.

A simplified version of manual lymph drainage aimed at stimulating lymph flow in unaffected areas of the body (neck, chest, and abdomen) was recommended for 20 min daily. In the CG, SLD was to be performed prior to donning the compression stockings.

### 2.3. Measurements

Measurements, including limb volume, weight-adjusted volume change (WAC), and disease-related symptoms, were performed preoperatively and at 3, 6, and 12 months postoperatively in both groups.

Limb volume assessment was conducted using circumference measurements taken with a standard measuring tape at 4 cm intervals applied without additional tension, starting from the lateral malleolus and extending to the hip joint. All measurements were performed in the morning, after a minimum of 12 h without compression (overnight break), to minimize the transient mechanical influence of compression on soft tissue volume. Lower leg volume was calculated using the simplified frustum (truncated cone) volume formula [14]. Additionally, foot circumference was measured at the level of the base of the metatarsal bones at the dorsum.

To account for interlimb differences and body weight fluctuations, the weight-adjusted volume change (WAC) formula was additionally applied to correct BMI changes over time [15].

Lymphedema-related symptoms—including pain, heaviness, skin tightness, limb numbness, genital edema, and lymphorrhea—were assessed using a 5-point Likert scale (0 = never; 4 = always).

Compression compliance in the CG was evaluated using the International Compression Club—Compression Questionnaire (ICC—CQ) [16], which considers both the functional status prior to compression initiation and the perception of the compression garments after 12 months. Symptom severity prior to compression use was assessed using a numeric rating scale (NRS) from 0 to 10 (0 = no symptoms; 10 = worst possible severity).

Compression comfort was evaluated after 12 months and included parameters such as ease of donning/doffing, sensations immediately after donning and during daily use, ease of wearing clothing over compression garments, and the appearance of the garment. Each domain was scored on a 0–10 NRS (0 = not able at all; 10 = completely able), and the scores were summed.

Compression-related side effects (e.g., skin irritation, tenderness, skin lesions, itching, warmth, throbbing, cramping, cutting in, slippage, localized swelling, bulky sensation, or excessive tightness) were rated on a 0–10 NRS (0 = not present at all; 10 = very obvious), with total scores calculated.

Health-related quality of life was assessed using the European Organization for Research and Treatment of Cancer Quality of Life Questionnaire (EORTC QLQ-C30), administered at 12 months postoperatively in both groups.

Subcutaneous edema was evaluated using the pitting test, with a positive result recorded when pressure applied to the skin produced a measurable indentation:

- Grade 1 = 2 mm;
- Grade 2 = 3–4 mm;
- Grade 3 = 5–6 mm;
- Grade 4 = ≥8 mm.

Lymphedema diagnosis was confirmed if lower leg volume increased by ≥10% between two subsequent assessments or by ≥8% in conjunction with a positive pitting sign.

### 2.4. Statistics 

Baseline demographic data were summarized using descriptive statistics: means with standard deviations (SDs) for normally distributed variables and medians with interquartile ranges (IQR) for non-normally distributed ordinal or quantitative variables, as assessed using the Shapiro–Wilk test. Continuous variables were summarized using medians and interquartile ranges (IQRs) due to the non-normal distribution of data.

Between-group comparisons for non-normally distributed variables were performed using the Mann–Whitney U test. Within-group comparisons of non-normally distributed variables assessed at two time points were also analyzed using the Mann–Whitney U test. For variables measured more than twice within a single group, the Friedman test was applied, followed by Wilcoxon signed-rank tests with Bonferroni correction for post hoc analysis.

A *p*-value of <0.05 was considered statistically significant. All statistical analyses were conducted using R statistical software (version 4.2.2, Vienna, Austria).

### 2.5. The Sample Size

The sample size was determined based on data from a previous pilot study. It was assumed that the incidence of lower limb lymphedema in women treated for reproductive tract malignancies would be approximately 40%. The use of prophylactic compression stockings was expected to reduce the incidence to 5%. Based on these assumptions, and using a type I error (α) of 0.05 and a type II error (β) of 0.20 (power = 80%), the minimum required sample size was calculated to be 42 participants (21 per group).

This study was conducted in accordance with the principles of the Declaration of Helsinki. The research protocol was approved by the Committee of Medical Ethics at the District Medical Chamber in Kraków (Approval No. 5/KBL/OIL/2016).

## 3. Results 

### 3.1. Limb Volumes and WAC

In the CG, a reduction in both right and left lower limb volumes was observed at 3, 6, and 12 months postoperatively. In contrast, the NCG demonstrated an increase in limb volume over the same period. The between-group difference in volume change was statistically significant (*p* = 0.001).

The incidence of unilateral LLL was significantly lower in the CG, affecting only 3.4% of participants (1/29), compared to 38.7% (12/31) in the NCG (*p* = 0.003). In the NCG, limb lymphedema was observed in two women at 3 months, six women at 6 months, and an additional four women at 12 months postoperatively. This structured follow-up allowed us to differentiate between transient postoperative edema and more persistent lymphedema over time.

Weight-adjusted volume change (WAC) values were consistently lower in the CG than in the NCG at all assessed time points—3, 6, and 12 months post-surgery (*p* = 0.001; see Table 2 for details) (Data are presented as n for categorical variables and as median (IQR) for continuous variables).

Participants diagnosed with lymphedema were excluded from further follow-up assessments in the study.

**Figure 2:**
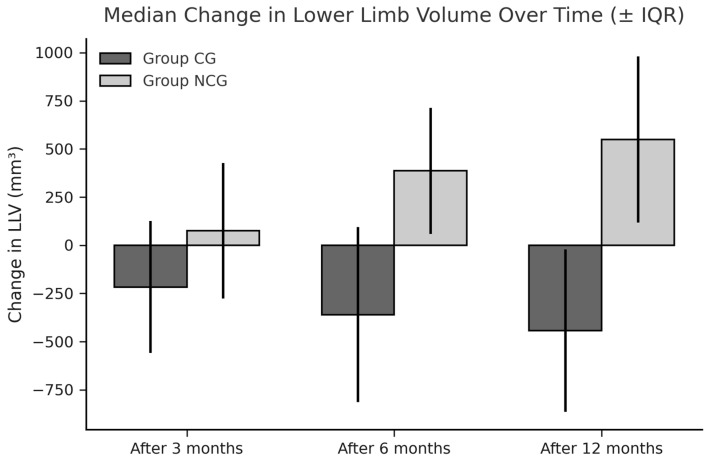
The median change in lower limb volume (LLV) over time within the analyzed groups.

The median leg volumes (mL) at baseline and 12 months post-surgery are shown in Figure 2. A progressive reduction in LLV was observed in the CG, while an increase was noted in the NCG. 

### 3.2. The Pitting Test

A positive pitting skin sign was observed in eight women (27%) from the NCG. Of these, seven patients were classified as grade 1, and one patient as grade 2. The onset of this symptom occurred in two women at 3 months post-surgery, in three women at 6 months, and in two women at 12 months.

In six of these patients, the lower leg volume increased by 9% and coexisted with a positive pitting sign, suggesting early-stage lymphedema. In all cases, the swelling was localized around the ankles or the distal parts of the lower legs.

In the CG, no positive pitting skin sign was observed 12 months postoperatively.

### 3.3. Compliance 

We specified that 29 out of 31 patients in the compression group reported full adherence to wearing the compression garments as instructed. Two patients reported partial or inconsistent use and were excluded from the final analysis due to protocol deviation. Among the adherent participants, compression stockings were worn for an average of 6.5 days per week, with a daily wear time of 9 h (range: 5–12 h). After the 12-month follow-up period, participants chose to continue wearing compression garments voluntarily based on personal comfort or perceived benefit.

Although most participants did not engage in structured sports activities (n = 22), the majority reported being physically active (n = 28). The most common forms of non-sport physical activity included

- Walking more than 1 km per day (n = 27);
- Nordic walking (n = 17);
- Cycling (n = 11);
- Gymnastic exercises (n = 9).

Women rated the ease of using compression stockings at an average of 9.6 out of 10. Most patients (n = 27) used assistive tools for donning and doffing, such as

- Rubber gloves (n = 24);
- Slippery socks or foot aids (n = 15);
- Assistance from another person (n = 15).

The ability to put on shoes over the stockings was rated at 9.9/10, and the ability to dress was rated at 9.8/10.

Comfort regarding wearing compression stockings was given

- A rating of 8.4/10 immediately after donning;
- A rating of 8.6/10 later in the day.

The visual appearance of the stockings received an average score of 8.2/10.

### 3.4. Health-Related Quality of Life and Disease-Related Symptoms

An improvement in symptoms such as pain, limb heaviness, skin tension, and limb numbness was reported in the CG beginning at 3 months postoperatively and persisting thereafter (*p* = 0.001). In contrast, patients in the NCG experienced worsening in the severity of these symptoms over time (Table 3). The data are presented as the median (IQR) for continuous variables and as the mean ± SD.

An analysis of the EORTC QLQ-C30 questionnaire data revealed no significant differences between the compression group (CG) and the no-compression group (NCG) in global health status, symptom scales, or most functional scales at any assessment point. However, physical functioning was significantly better in the CG 12 months after surgery (*p* = 0.03) (Table 4).

## 4. Discussion

Several studies have demonstrated that compression therapy is an effective treatment modality [17,18] and a cost-effective strategy for preventing recurrent cellulitis in patients with chronic edema [19]. However, scientific evidence specifically addressing the prevention of gynecological cancer-related lymphedema remains limited.

Although the beneficial physiological effects of compression therapy on lymphatic function—such as reducing pressure in initial lymphatic vessels, limiting interstitial fluid filtration, enhancing lymphatic reabsorption, stimulating lymphangion contractions, and exerting anti-inflammatory effects via activation of the parasympathetic (vagal) system—have been well documented [20,21,22,23], prophylactic compression-based physiotherapy has not yet been adopted as a standard of care for gynecological cancer survivors.

Most of the current literature on secondary lymphedema prevention has focused on the upper limbs in the context of breast cancer surgery [24,25,26,27]. In contrast, considerably less is known about the prevention of LLL following treatment for gynecologic malignancies. Notably, prospective randomized controlled trials evaluating the long-term effects of postoperative compression therapy—especially when initiated preoperatively and combined with structured exercise—are still lacking in this patient population.

To the best of our knowledge, this is the first prospective randomized study to demonstrate the effectiveness of medium-pressure compression stockings (23–32 mmHg), combined with physical exercise and patient education, administered over a one-year period, in the prevention of early LLL in women undergoing gynecological cancer surgery.

Patients in the compression group experienced significant reductions in limb volume and symptom severity compared to the control group, indicating the potential of this multifaceted approach as an effective preventive strategy.

Our findings highlight the importance of early implementation of compression therapy as part of preventive care and stand in contrast to findings of prior studies in this field.

For example, a small pilot study by Sawan et al. involving seven patients with vulvar cancer reported a greater mean increase in leg volume among patients who did not wear compression stockings compared to those who used them for six months; however, the difference was not statistically significant [11].

Similarly, Shallwani et al. found no significant difference in the incidence of clinically diagnosed lymphedema between groups. Nevertheless, the mean leg volume increased more in the control group, and exploratory data suggest a potential delay in lymphedema onset in the intervention group using compression therapy [12].

In contrast, our study demonstrated a significantly lower incidence of lower limb lymphedema: 3.4% (1/29) in the compression group versus 38.7% (12/31) in the control group (*p* = 0.003). These results suggest that an early, structured intervention combining medium-pressure compression stockings, education, and physical activity may offer substantial preventive benefits for gynecological cancer survivors.

No significant benefit from medium-pressure compression stockings (23–32 mmHg, class II) in the prevention of LLL was observed in the study by Stuiver et al. [13]. A major limitation of that study was the heterogeneity of the patient population, which included individuals with melanoma and various urogenital cancers at different stages of clinical progression.

Unexpected findings were also reported by Zhang et al., who found that progressive resistance exercise training (PRET) was more effective than graduated compression stockings (GCSs; 15–20 mmHg) in preventing LLL after pelvic lymphadenectomy for cervical cancer [28]. The PRET group had a significantly lower risk of developing lymphedema (*p* < 0.001), whereas the GCS and control groups showed only a non-significant protective trend. In that study, patients were instructed to wear GCSs for at least 23 h per day, which led to frequent dissatisfaction, especially during summer months, due to symptoms such as pruritus, heat, or blistering. These issues suggest that poor adherence—likely related to discomfort or inappropriate use—may have affected outcomes. Furthermore, the data raise questions about the necessity of continuous 23 h compression, particularly given that compression is most effective during daytime activity, when lymphatic flow is enhanced by movement.

Similarly, Woods et al. concluded that prophylactic compression stockings did not significantly reduce the development of lymphedema [29]. However, the study lacked reliable data on patient compliance, making it unclear whether compression was used consistently or as prescribed.

Several factors have been identified as contributing to non-adherence with compression therapy, including discomfort, pain, poor fit or esthetics, difficulty with donning and doffing, and seasonal discomfort related to heat. Environmental factors such as high temperature may also negatively impact patient compliance.

The results of our study contrast with previous research not only in terms of the lower incidence of lymphedema in the compression group, but also with respect to the duration of compression use and high patient compliance. Participants in our study wore compression stockings for an average of 6.5 days per week, 9 h per day, over a 12-month period and reported high levels of satisfaction. The extended duration of compression use appears justified as lymphedema most commonly develops within the first year after oncologic treatment—or even later. 

Notably, the observed reduction in limb volume among participants in the compression group may reflect multiple physiological mechanisms. Beyond the prevention of lymphedema-related swelling, this decrease could also be attributed to the resolution of transient postoperative edema, enhanced lymphatic drainage, or improved venous return associated with compression therapy. While these findings support the beneficial role of compression, they also raise the important question of potential overtreatment in asymptomatic patients. In this study, however, the reductions in limb volume remained within physiological ranges, and no side effects were reported, suggesting that the intervention was well tolerated. Nevertheless, future studies should explore individualized compression protocols to balance efficacy with the risk of unnecessary intervention in low-symptom or subclinical populations. Beyond reducing objective limb volume, compression therapy also alleviated subjective symptoms of lower limb discomfort, contributing to improved perceived quality of life. Importantly, compression use did not exacerbate any symptoms; on the contrary, it significantly reduced the severity of multiple complaints. These findings suggest that compression stockings not only fail to reduce well-being, as sometimes feared, but may in fact enhance certain dimensions of patient comfort and daily functioning.

Health-related quality of life (HRQoL) assessed at 12 months was comparable between the two groups, with the exception of physical functioning, which was significantly better in the compression group. This difference may be explained by greater confidence in engaging in physical activity among compression wearers, who were likely less concerned about triggering lymphedema and more able to maintain regular domestic and daily living tasks.

At present, there is no consensus regarding the optimal method for detecting subclinical or early-stage lymphedema, and diagnostic criteria remain non-standardized. The variability in assessment tools and thresholds used across studies may contribute to delayed intervention and limit the comparability of findings across research settings [5]. In our study, we followed international guidelines defining subclinical lymphedema as a volume difference of ≥10%. However, we acknowledge that this threshold remains subject to debate. In cases where pitting (soft, pliable) edema was observed, a volume difference of ≥8% was considered sufficient to establish a diagnosis. 

Our prospective surveillance model—incorporating circumferential measurements, symptom monitoring, and skin assessments—proved to be both reliable and practical for the early detection of lower limb lymphedema. Circumferential tape measurement was selected as an inexpensive, reproducible, and widely accessible method suitable for routine clinical use [30].

In addition, we utilized the weight-adjusted volume change (WAC) formula, originally developed to quantify arm volume changes in patients following bilateral mastectomy and to identify risk factors for breast cancer-related lymphedema (BCRL) [15]. This method takes into account individual limb volume fluctuations and overall body weight changes, offering a more personalized assessment.

In our study, WAC values significantly decreased in the compression group at 12 months post-surgery, further supporting the beneficial effect of medium-pressure compression therapy as part of a preventive strategy.

The use of the WAC formula may be particularly valuable for quantifying gynecological cancer-related lymphedema, especially in patients undergoing bilateral inguinofemoral lymphadenectomy and pelvic radiotherapy. This method offers a sensitive and individualized approach to early detection by accounting for both limb-specific changes and overall body weight fluctuations.

However, the limited number of studies utilizing the WAC formula in this specific patient population makes direct comparison with the existing literature challenging. Further research is warranted to validate its clinical utility and to establish standardized thresholds for the early detection and monitoring of lower limb lymphedema in women treated for gynecologic malignancies.

Another important aspect concerns the pressure exerted by compression garments in patients at risk of developing lymphedema. The optimal pressure range for compression stockings in the prevention of LLL remains an open question. According to the RAL-GZG standard classification, a pressure range of 23–32 mmHg (medium compression, class II) is considered therapeutic, particularly in the treatment of lymphedema and chronic venous disease [23].

Previous studies have shown that even low-pressure compression (18–21 mmHg) may be effective in preventing upper limb lymphedema in breast cancer survivors [25]; however, this pressure level appears to be insufficient for gynecological cancer survivors, as indicated in the studies by Sawan and Shallwani [11,12]. In healthy women, compression stockings exerting 18–29 mmHg at the ankle and 15–23 mmHg at the calf have been shown to reduce leg edema [31]. Based on these observations, the pressure range of 23–32 mmHg used in our study was considered optimal.

On the one hand, this medium pressure was effective in preventing postoperative swelling and lymphedema. On the other hand, it was also well tolerated and did not negatively impact comfort or quality of life in our patients. Compression may improve fluid balance by reducing capillary filtration and enhancing fluid reabsorption into both the venous and lymphatic systems, yet its role as a risk reduction strategy for lymphedema remains incompletely understood [32].

Physical exercise may further promote lymphatic drainage and has been shown to be safe for individuals with or at risk of lymphedema. However, current evidence does not confirm a direct effect of exercise alone on reducing limb volume in at-risk patients [33]. Nevertheless, there is broad agreement that physical activity plays a critical role in the health of gynecological cancer survivors. The American College of Sports Medicine recommends exercise as a safe and beneficial intervention during and after cancer treatment. Regular physical activity may improve overall survival, reduce the risk of recurrence, and lower cancer-related mortality [34].

The benefits of exercise in gynecologic cancer survivors have been well documented and include reduced fatigue, improved physical function, better weight management, and enhanced quality of life (QoL) [33,35].

A systematic review and meta-analysis have demonstrated that various forms of exercise—including active, aerobic, and aquatic training—do not exacerbate gynecological cancer-related lymphedema and can therefore be safely recommended for women following treatment for gynecological malignancies [32]. The duration of exercise sessions may vary from 15 to 50 min depending on individual health status and tolerance.

However, there is currently no consensus on whether physical activity should be performed with or without compression garments in patients at risk of developing LLL. Some studies suggest that wearing compression stockings or bandages during exercise may maximize the reduction in lower limb swelling. In fact, this combined approach has been shown to be more effective than compression therapy alone in gynecological cancer survivors with established LLL [36,37].

A synergistic mechanism has been proposed to explain this enhanced effect. When used during exercise, compression garments may improve lymphatic valve function, support lymphatic vessel contractility, promote fluid reabsorption, and limit excess interstitial fluid accumulation [21].

It has been suggested that active limb exercise performed under appropriate compression (20–60 mmHg) may serve as an effective conservative treatment for lymphedema and can be safely implemented as a daily routine [17]. In our study, 20 min of daily walking while wearing medium-pressure compression stockings (23–32 mmHg) was shown to be highly beneficial in improving physical function.

This simple, low-cost intervention may represent a practical and effective preventive strategy for gynecological cancer survivors at risk of developing lower limb lymphedema.

Limited evidence also supports the role of health education [38] and the early application of Complex Decongestive Therapy (CDT) [1,9,10] in reducing the incidence of lower limb lymphedema (LLL) in patients following gynecological cancer surgery. According to the consensus of the International Society of Lymphology, CDT is primarily recommended for patients with established lymphedema.

One of the core components of CDT—compression bandaging—has been shown to be highly effective in reducing limb volume, as reported in the study by Wu et al. [1]. However, this method may be less suitable for long-term prophylactic use in at-risk populations due to its complexity and limited practicality in routine self-management.

Although compression therapy is often promoted as a preventive measure, it is more accurate to state that it reduces or delays the onset of clinically visible lymphedema-related swelling. It does not prevent the underlying lymphatic dysfunction but may limit progression to symptomatic stages when initiated early and applied consistently.

Although the pitting test was used to support the clinical diagnosis of lymphedema, it should be acknowledged that this method lacks complete specificity and may yield positive results in other forms of edema, such as lipedema or generalized fluid retention. In contrast, the skinfold thickness test is considered a more specific diagnostic tool, particularly for distinguishing between lymphedema and lipedema. Unfortunately, due to resource limitations, this assessment could not be implemented during the study period. Future studies should consider incorporating this parameter to improve diagnostic precision and strengthen clinical evaluation of lymphedema.

Importantly, patient adherence is a key determinant of success in preventive physiotherapy programs. In our study, participants successfully learned proper donning and doffing techniques for compression stockings and were able to perform SLD with relative ease.

The combined strategy of structured health education, regular monitoring, and early compression-based physiotherapy yielded promising outcomes. This integrative approach appears to be both feasible and effective for gynecological cancer survivors at risk of developing lower limb lymphedema.

Although compression therapy is often promoted as a preventive measure, it is more accurate to state that it reduces or delays the onset of clinically visible lymphedema-related swelling. It does not prevent the underlying lymphatic dysfunction but may limit the progression to symptomatic stages when used early and consistently.

### Limitations and Strengths

One major limitation of our study is the relatively small sample size, which limited the ability to perform subgroup analyses related to specific risk factors for lymphedema development. Additionally, nine patients were excluded after surgical verification—three due to postoperative complications and six due to cancer recurrence or metastasis. Furthermore, two participants withdrew from the study, citing general discomfort, lack of consent for 12-month compression use, or unwillingness to continue participation.

Another limitation is the relatively short follow-up period. Previous research has shown that a substantial proportion of patients develop lower limb lymphedema more than one year postoperatively. Therefore, future studies with longer follow-up durations are needed to evaluate the long-term efficacy of compression therapy combined with health education in gynecological cancer survivors.

One of the limitations of this study is the absence of skinfold thickness assessment, which is considered a more specific method for detecting lymphedema compared to clinical tests such as the pitting test. At the time of data collection, the necessary equipment and training for this measurement were not available in our setting.

A limitation of our study is the lack of a discontinuation phase to assess potential rebound swelling following cessation of compression therapy. As such, we were unable to evaluate whether the beneficial effects of compression were sustained after stopping the intervention or whether continued use is necessary to maintain volume reduction. This highlights the need for future studies to include structured withdrawal or follow-up protocols to better understand the long-term impact and optimal duration of compression in at-risk populations.

Despite these limitations, this study has several important strengths. The use of a homogeneous patient population, along with high adherence to compression therapy—both in terms of correct application and consistent daily use—contributed to the reliability of the findings. Participants also demonstrated regular follow-up attendance, enhancing data completeness.

Moreover, the study offers direct clinical relevance. Its findings suggest a practical, low-cost, and easily implementable strategy for preventing lower limb lymphedema in gynecological cancer survivors—one that may be readily integrated into routine oncologic care.

## 5. Conclusions

Compression stockings exerting a pressure range of 23–32 mmHg, when combined with regular physical activity and structured health education, represent a safe, well-tolerated, and effective strategy for reducing or delaying post-surgical swelling and the onset of visible LLL between 3 and 12 months following radical oncological treatment for gynecologic cancer.

Future clinical trials are warranted to further optimize compression protocols and to establish standardized, evidence-based recommendations for the prevention of LLL in at-risk gynecological cancer survivors.

## Figures and Tables

**Table 1:** Baseline characteristics of enrolled patients.

Parameter (n or Median, IQR)	CG (n = 29)	NCG (n = 31)	*p*-Value
Age (years)	53.5 (15.25)	58 (14.0)	0.06 *
Height (m)	1.61 (0.11)	1.58 (0.09)	0.3 *
Weight (kg)	70.0 (16.5)	75.0 (19.0)	0.9 *
Body mass index (kg/m^2^)	26.9 (4.6)	28.0 (8.6)	0.5 *
Professionally active	19	16	
Tumor localization (n)			
Ovaries	7	7	0.6 **
Cervix uteri	8	8	
Corpus uteri	12	15	
Vulva	2	1	
Complementary therapy (n)			
Lymphadenectomy	29	31	
Radiotherapy	26	26	0.9 **
Chemotherapy	13	14	
Limb volumes (L)			
Right	8.13 (1.36)	7.82 (2.28)	0.8 *
Left	8.30 (1.93)	7.90 (2.17)	0.9 *
Right and left	16.60 (3.58)	15.95 (4.38)	0.9 *

* Mann–Whitney test; ** Fisher’s exact test. IQR, interquartile range; CG, compression group; NCG, no-compression group.

**Table 2:** Edema occurrence and volume changes (mL) within the analyzed groups.

Parameter (n or Median, IQR)	CG (n = 29)	NCG (n = 31)	*p*-Value
Edema occurrence (n)	1	12	0.003 *
Limb volume changes			
* Right limb*			
Within 3 months	−86.13 (349.40)	97.05 (315.27)	0.049 **
Within 6 months	−157.33 (480.43)	193 (836.04)	0.022 **
Within 12 months	−259.33 (540.99)	250.05 (546.81)	0.001 **
*p*-value	0.01 ***	0.07 ***	
* Left limb*			
Within 3 months	−155.66 (285.45)	36.80 (388.09)	0.051 **
Within 6 months	−198.42 (94.43)	151.37 (384.09)	0.019 **
Within 12 months	−137.10 (497.68)	250.51 (366.36)	0.002 **
*p*-value	0.011 ***	0.06 ***	
* Right and left limb*			
Within 3 months	−216.88 (684.90)	75.34 (162.36)	0.032 **
Within 6 months	−359.81 (375.47)	386.96 (655.08)	0.004 **
Within 12 months	−443.01 (841.88)	549.07 (861.70)	0.001 **
*p*-value	0.011 ***	0.011 ***	
WAC			
* Right limb*			
Within 3 months	−0.01 (0.04)	0.02 (0.06)	0.004 **
Within 6 months	−0.03 (0.06)	0.03 (0.02)	0.003 **
Within 12 months	−0.06 (0.08)	0.02 (0.05)	0.001 **
*p*-value	0.001 ***	0.9 ***	
* Left limb*			
Within 3 months	−0.02 (0.05)	0.01 (0.05)	0.002 **
Within 6 months	−0.03 (0.07)	0.02 (0.05)	0.003 **
Within 12 months	−0.05 (0.07)	0.02 (0.05)	0.001 **
*p*-value	0.001 ***	0.3 ***	
* Right and left limb*			
Within 3 months	−0.02 (0.03)	0.02 (0.03)	0.001 **
Within 6 months	−0.03 (0.05)	0.02 (0.03)	<0.001 **
Within 12 months	−0.05 (0.06)	0.02 (0.04)	<0.001 **
*p*-value	0.001 ***	0.4 ***	

* Chi square test; ** Mann–Whitney test; *** Friedman test with post hoc analysis. WAC, weight-adjusted volume change; IQR, interquartile range, CG, compression group; NCG, no-compression group.

**Table 3:** Symptom burden within the analyzed groups.

Problem Mean ± SD and Median (IQR)	CG (n = 29)	*p*-Value Within Groups **	NCG (n = 31)	*p*-Value Within Groups **	*p*-Value Between Groups *
Pain within the limbs					
At baseline	1.39 ± 0.57 1.0 (1.0)		1.33 ± 0.61 1.0 (0.75)		**0.049**
After 3 months	1.41 ± 0.57 1.0 (1.0)	1.0	1.50 ± 0.63 1.0 (1.0)	0.07	0.6
After 6 months	1.29 ± 0.53 1.0 (0.25)	0.2	1.57 ± 0.63 1.5 (1.0)	**0.04**	0.6
After 12 months	1.32 ± 0.55 1.0 (1.0)	0.5	1.59 ± 0.68 1.0 (1.0)	**0.05**	0.1
Limb heaviness					
At baseline	1.26 ± 0.39 1.0 (0.5)		1.23 ± 0.43 1.0 (0.0)		0.8
After 3 months	1.33 ± 0.48 1.0 (1.0)	0.5	1.27 ± 0.52 1.0 (0.0)	0.8	0.5
After 6 months	1.33 ± 0.48 1.0 (1.0)	0.5	1.73 ± 0.74 2.0 (1.0)	**0.004**	**0.036**
After 12 months	1.54 ± 0.58 1.5 (1.0)	0.06	1.62 ± 0.68 2.0 (1.0)	**0.003**	0.7
Skin tension					
At baseline	1.18 ± 0.41 1.0 (0.0)		1.23 ± 0.43 1.0 (0.0)		0.7
After 3 months	1.28 ± 0.46 1.0 (1.0)	0.5	1.43 ± 0.50 1.0 (1.0)	**0.04**	0.3
After 6 months	1.43 ± 0.57 1.0 (1.0)	0.2	1.60 ± 0.67 1.0 (1.0)	**0.003**	0.1
After 12 months	1.43 ± 0.57 1.0 (1.0)	0.008	1.48 ± 0.63 1.0 (1.0)	**0.04**	0.8
Limb numbness					
At baseline	1.64 ± 0.68 1.0 (1.0)		1.47 ± 0.50 1.0 (1.0)		0.3
After 3 months	1.46 ± 0.58 1.0 (1.0)	0.09	1.70 ± 0.65 2.0 (1.0)	**0.02**	0.2
After 6 months	1.43 ± 0.50 1.0 (1.0)	**0.01**	1.73 ± 0.64 2.0 (1.0)	**0.04**	**0.04**
After 12 months	1.43 ± 0.50 1.0 (1.0)	0.09	1.69 ± 0.60 2.0 (1.0)	0.08	
Genital edema					
At baseline	1.00 ± 0.00 1.0 (0.0)		1.00 ± 0.00 1.0 (0.0)		1.0
After 3 months	1.08 ± 0.27 1.0 (0.0)	0.3	1.07 ± 0.25 1.0 (0.0)	0.3	0.9
After 6 months	1.00 ± 0.00 1.0 (0.0)	1.0	1.17 ± −0.46 1.0 (1.0)	0.09	**0.049**
After 12 months	1.00 ± 0.00 1.0 (0.0)	1.0	1.07 ± 0.26 1.0 (0.0)	0.4	0.1
Lymphorrhea					
At baseline	1.00 ± 0.00 1.0 (0.0)		1.00 ± 0.00 1.0 (0.0)		1.0
After 3 months	1.00 ± 0.00 1.0 (0.0)	1.0	1.03 ± 0.18 1.0 (0.0)	1.0	0.4
After 6 months	1.00 ± 0.00 1.0 (0.0)	1.0	1.13 ± 0.43 1.0 (0.0)	0.2	0.09
After 12 months	1.00 ± 0.00 1.0 (0.0)	1.0	1.00 ± 0.00 1.0 (0.0)	1.0	1.0

* Mann–Whitney U test; ** *p*-value according to baseline, Wilcoxon paired test; IQR, interquartile range.

**Table 4:** Quality of life according to EORTC QLQ-C30 questionnaire after 12 months.

Parameter Median (IQR)	CG (n = 29)	NCG (n = 31)	*p*-Value
Global health status	75 (25.0)	66.67 (16.67)	0.3
Physical functioning	93.33 (13.33)	86.67 (20.0)	**0.03**
Role functioning	100 (20.83)	83.33 (16.67)	0.8
Emotional functioning	83.33 (25.0)	75.0 (25.0)	0.9
Cognitive functioning	91.67 (33.33)	83.33 (16.67)	0.5
Social functioning	100 (16.67)	100 (16.67)	0.9
Fatigue	22.22 (33.33)	22.22 (22.22)	0.5
Nausea and vomiting	0.0 (0.0)	0.0 (12.5)	0.8
Pain	16.67 (33.3)	25.0 (33.33)	0.3
Dyspnea	0.0 (0.0)	0.0 (33.33)	0.2
Insomnia	33.33 (33.33)	33.33 (33.33)	0.9
Appetite loss	0.0 (0.0)	0.0 (33.33)	0.4
Constipation	16.67 (33.33)	0.0 (33.33)	0.4
Diarrhea	0.0 (0.0)	0.0 (0.0)	0.4
Financial difficulties	0.0 (33.3)	0.0 (33.3)	0.6

Comparison between groups (Mann–Whitney U test). IQR, interquartile range; EORTC, European Organization for Research and Treatment of Cancer; CG, compression group; NCG, no-compression group.

## Data Availability

The data that support the findings of this study are available from the corresponding author upon reasonable request.
